# NCR, an Inflammation and Nutrition Related Blood-Based Marker in Colon Cancer Patients: A New Promising Biomarker to Predict Outcome

**DOI:** 10.3390/diagnostics13010116

**Published:** 2022-12-30

**Authors:** Melanie Langheinrich, Alexander Reinhard Siebenhüner, Justus Baecker, Maximilian Miragall, Felix Wiesmüller, Vera Schellerer, Susanne Merkel, Maximilian Brunner, Christian Krautz, Klaus Weber, Robert Grützmann, Stephan Kersting

**Affiliations:** 1 Department of Surgery, University Hospital of Greifswald, 17475 Greifswald, Germany; 2 Department of Gastroenterology and Hepatology, University Hospital Zurich and University Zurich, 8006 Zurich, Switzerland; 3 Department of Surgery, University Hospital of Erlangen, 91054 Erlangen, Germany

**Keywords:** colorectal cancer, colon cancer, biomarker, tumor marker, inflammation, nutrition, sidedness, prognosis

## Abstract

Background: Colorectal carcinoma (CRC) is a heterogeneous disease, and differences in outcomes have been reported among patients diagnosed with the same disease stage. Prognostic and predictive biomarkers provide information for patient risk stratification and guide treatment selection. Although numerous studies have analyzed the effects of systemic inflammatory factors on CRC outcomes, clinical significance remains to be elucidated. In particular, the treatment strategy of colon cancer patients is different from that of rectal cancer due to outcome and recurrence differences. The identification of patients with a poor prognosis who might benefit from intensive treatment approaches is clinically necessary. Methods: This study aimed to evaluate the value of different blood-based markers and assess the significance of our newly developed inflammatory-nutrition-related biomarker (NCR = BMI × albumin/CRP) in patients with colon cancer. A two-stage design was used with 212 patients with colon cancer (CC) in the discovery cohort (*n* = 159) and in an external validation cohort (*n* = 53). Results: A lower preoperative NCR level was significantly correlated with a worse prognosis, sidedness, undifferentiated histology, nodal involvement, and advanced UICC stage. We compared the NCR with other established prognostic indices and showed that the NCR is a more reliable indicator of a poor prognosis for patients with CC. Patients with low NCR levels experienced a significantly shorter Overall Survival (OS) than patients with high levels. Multivariate analysis confirmed preoperative NCR levels as an independent predictor for overall survival with a hazard ratio of 3.3 (95% confidence interval 1.628–6.709, *p* < 0.001). Finally, we confirmed the predictive value of the NCR in an independent validation cohort and confirmed NCR as an independent prognostic factor for OS. Conclusion: Taken together, we discovered a new prognostic index (NCR) based on BMI, albumin, and CRP levels as an independent prognostic predictor of OS in patients with colon cancer. In all UICC stages, our newly developed NCR marker is able to distinguish patients with better and worse prognoses. We, therefore, propose that NCR may serve as a supplement to the TNM staging system to optimize the risk stratification in CC patients towards personalized oncology. In particular, NCR can be used in clinical trials to stratify patients with UICC II and III tumors and help better select patients who might benefit from adjuvant treatment.

## 1. Introduction

Colorectal cancer (CRC) is the third most frequently diagnosed cancer and the second most common cause of cancer-related death worldwide [1,2]. Tumor location (right- and left-sided colon cancer, rectal cancer) is widely accepted as a crucial factor determining disease progression and prognosis and influences disease management [3]. While the prognosis of patients with early-stage disease is excellent, ultimately, 40% of patients across all disease stages die from their disease within five years [4]. The prognosis of CRC is principally related to the tumor, node, and metastasis (TNM) stage. Additionally, several clinicopathological factors have been identified as predictive of outcomes. However, the TNM stage system is the gold standard for treatment selection and outcome prediction, with significant limitations. The system lacks the ability to predict patient outcomes individually. Currently, adjuvant chemotherapy is not routinely recommended for patients with UICC stage II CC. It is recommended for patients with high-risk factors, such as T4 tumors, bowel obstruction, perforation, lymphovascular invasion, or poorly differentiated tumors [5,6]. Little evidence is available that patients with high-risk factors benefit from adjuvant chemotherapy compared with patients without these high-risk factors. In the era of precision oncology, predicting survival is essential since more radical treatment approaches might reduce the quality of life. Therefore, the identification of easily accessible, convenient, practical, and preoperatively available biomarkers that help identify patients at higher risk of poor outcomes is necessary.

Carcinoembryonic antigen (CEA) is the most accepted and routinely used colorectal tumor marker for screening, predicting treatment response and survival, and detecting recurrence [7]. Preoperative CEA levels have been reported to be positive in only 40–60% of patients at the initial diagnosis, and the usefulness of CEA monitoring for reducing CRC mortality in postoperative patients or selecting those patients with stage II tumors who would benefit from adjuvant chemotherapy is controversial [8,9]. Obesity is considered to be a risk factor for various types of cancer. The patient’s nutritional status is related to cancer prognosis and survival, such as body mass index (BMI), prognostic nutritional index (PNI, calculated by adding factorized albumin levels and lymphocyte counts), advanced lung cancer inflammation index (ALI, consisting of BMI, serum albumin levels and NLR), the C-reactive protein (CRP)-to-albumin ratio (CAR), and the CONUT score [10,11,12,13,14]. In addition to the patient’s nutritional status, many studies have shown that the host systemic inflammatory response (SIR), measured by inflammation-based scores, such as the lymphocyte-to-C-reactive protein ratio (LCR), neutrophil-to-lymphocyte ratio (NLR), and platelet-to-lymphocyte ratio (PLR), is a key factor in determining outcomes and survival [6,15,16,17].

However, due to the heterogeneity of CRC, the results of these studies are inconsistent. Up to now, colon cancer and rectal cancer patients are commonly considered as a single disease entity in these outcome studies. For this reason, we focused our attention on discerning blood-based markers governing colon cancer only. We further hypothesize that identifying parameters reflecting the host inflammation and nutritional status together with tumor characteristics may be a better approach for predicting patients’ overall survival at baseline. We defined a new marker NCR (Nutritional CRP Ratio) as BMI*albumin/CRP levels, combining nutritional status and the current inflammatory response. We tested the newly developed NCR marker for its association with overall survival in patients with colon cancer (CC) in our discovery cohort and an independent validation cohort. Our newly developed marker may supplement the TNM staging system to tailor treatment strategies and optimize the risk stratification in CC patients toward individualized treatment.

## 2. Materials and Methods

### 2.1. Patients

In this study, a two-stage design with discovery and validation cohorts of patients with colon cancer was used, and 212 treatment-naïve patients were enrolled. The discovery cohort consisted of 159 patients with CC who underwent elective surgery at the Department of Surgery in Erlangen, Germany, between 2005 and 2013. Rectal cancer patients and patients who needed emergency surgery were excluded. Data were collected prospectively, and patients were followed until death or January 2017. Routine follow-up was performed at three- and six-month intervals for the initial two years and yearly thereafter for a total of five years. No patients were lost during follow-up. The tumor stage was determined according to the eighth edition of the TNM classification of the Union Internationale Contre le Cancer (UICC). We recruited 53 patients from the University Hospital Zürich, Switzerland, for the independent validation cohort. The study was approved by the respective local ethics committees, and all included patients signed informed consent forms.

### 2.2. Laboratory Measurements of Inflammatory-Related Blood Indices

Blood samples were collected before surgery (within one week) by peripheral venous puncture during a routine preoperative blood draw. The laboratory data included red and white blood cell counts and neutrophil, lymphocyte, platelet, albumin (g/L), CRP (mg/L), and CEA levels. Our newly developed NCR was calculated as the BMI*albumin to CRP ratio. Based on previous studies on inflammation-based prognostic biomarkers, we focused on five established markers: NLR, which is calculated by dividing the total neutrophil count by the total lymphocyte count; CAR, the CRP to albumin ratio; PLR, the platelet to lymphocyte ratio; LCR, the total lymphocyte count to CRP ratio; PNI, which is calculated as the albumin level + 0.005 × total lymphocyte count; and ALI, BMI*albumin/NLR. BMI scores were calculated as weight (kg)/height (m)^2^.

### 2.3. Laboratory Measurements of sICAM Concentrations Using ELISA

We previously evaluated sICAM (soluble intercellular adhesion molecule) [18]. Measurement was drawn from the same routine blood sample in S-Monovette 9 mL, Clotting Activator/Serum (Sarstedt, Nümbrecht, Germany, ref. 02.1063) and was allowed to clot for 30 min. After centrifugation at 2500× *g* for 10 min at room temperature, the supernatant was collected in aliquots and frozen at −80 °C until analysis. Soluble ICAM-1 levels were measured using the human ICAM-1/CD54 nonallele-specific Quantikine ELISA kit (Cat. no. SCIM00, R&D Systems, Inc., Minneapolis, MN, USA) according to the manufacturer’s instructions. Every blood sample was measured in duplicate.

### 2.4. Statistical Analysis

Statistical analyses were performed using the SPSS statistical software package version 27 (IBM SPSS Statistics v.27). A receiver operating characteristic (ROC) curve was generated to analyze the area under the ROC curve (AUC), and the Youden index was calculated to identify the optimal cutoff values (maximum sensitivity and specificity for all cutoff points in the ROC curve) for analyzing OS. For estimation of overall survival, we defined death from any cause as an event. Survival analysis was performed using the Kaplan–Meier (KM) method, and the log-rank test was used to compare the differences in survival. Univariate and multivariate Cox regression analyses were performed to calculate corresponding hazard ratios (HRs) and 95% confidence intervals (CIs). A *p*-value < 0.05 was considered statistically significant.

## 3. Results

### 3.1. Patients Characteristics

Among the discovery cohort of 159 patients included in the study, the median age was 66 years (range 27–91 years), with 103 males (64.8%) and 56 females (35.2%). No patients who needed emergency surgery were included. Thirteen patients (8.2%) had UICC I stage disease, 76 patients (47.8%) had UICC II stage tumors, 48 patients (30.2%) had UICC III stage tumors, and 22 patients (13.8%) had UICC stage IV disease. Baseline clinical and pathological characteristics are shown in Table 1. The validation cohort consisted of 53 patients with a median age of 68 years, including 29 males (54.7%) and 24 females (45.3%). No patients were diagnosed with UICC stage I tumors, 19 patients (35.9%) were diagnosed with UICC stage II tumors, 13 patients (24.5%) had UICC stage III disease, and 21 patients (39.6%) had UICC stage IV disease (Table 2).

### 3.2. Preoperative NCR Predicts Oncological Outcomes of Patients with Colon Cancer with Higher Accuracy Than Other Inflammation-Related Factors

**Figure 1:**
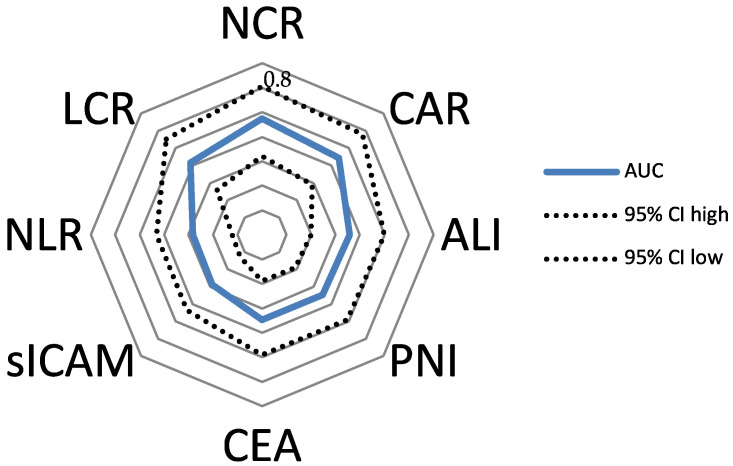
Side-to-side comparison of AUCs for different parameters predicting the OS of the discovery cohort.

The ROC curve analysis and Youden index indicated optimal cutoff values (Table 3, Figure 1, Supplementary Figure S1): NCR cutoff value was >255.13 at the highest Youden index of 0.48, with a sensitivity of 61.32% and specificity of 86.79% (AUC 0.737; 95% CI 0.661–0.803; *p* < 0.001). The CAR cutoff value was ≤0.11 at the highest Youden index of 0.44, with a sensitivity of 59.43% and a specificity of 84.91% (AUC 0.722; 95% CI 0.645 to 0.790; *p* < 0.001). The LCR cutoff value was >0.24 at the highest Youden index of 0.43, with a sensitivity of 66.04% and specificity of 77.36% (AUC 0.707; 95% CI 0.629–0.776; *p* < 0.001). The ALI cutoff value was >252.19 at the highest Youden index of 0.31, with a sensitivity of 83.96% and specificity of 47.17% (AUC 0.679; 95% CI 0.6–0.75; *p* < 0.001). The sICAM cutoff value was ≤229.1 ng/mL at the highest Youden index of 0.27, with a sensitivity of 46.23% and specificity of 81.13% (AUC 0.643; 95% CI 0.564–0.718; *p* < 0.002). The PNI cutoff value was >42.6 at the highest Youden index of 0.33, with a sensitivity of 65.09% and specificity of 67.92% (AUC 0.674; 95% CI 0.595–0.746; *p* < 0.001). The NLR cutoff value was ≤3.54 at the highest Youden index of 0.2925, with a sensitivity of 70.75% and specificity of 58.49% (AUC 0.641; 95% CI 0.561–0.716; *p* < 0.004) (Table 3). Accordingly, the patients were divided into a high NCR group (NCR > 255.13) and a low NCR group (NCR ≤ 255.13); high CAR group (CAR > 0.11) and low CAR group (CAR ≤ 0.11); high LCR group (LCR > 0.24) and low LCR group (LCR ≤ 0.24); high ALI group (ALI < 252.19) and low ALI group (ALI ≤ 252.19); high sICAM group (sICAM > 229.1 ng/mL) and low sICAM group (sICAM ≤ 229.1 ng/mL); high PNI group (PNI > 42.6) and low PNI group (PNI ≤ 42.6); and high NLR group (NLR > 3.54) and low NLR group (NLR ≤ 3.54). Although almost all of the evaluated markers significantly predicted shorter overall survival of patients with colon cancer, our newly developed marker, the preoperative NCR, predicted a shorter OS with the highest degree of accuracy. Based on these findings, we decided to focus this study on the NCR. Next, we evaluated the correlation between the NCR and clinicopathological findings.

### 3.3. Association between NCR and Clinicopathological Characteristics in the Discovery Cohort of Patients with Colon Cancer: A Decreased Preoperative NCR Correlated Significantly with Sidedness and the UICC Stage

**Figure 2:**
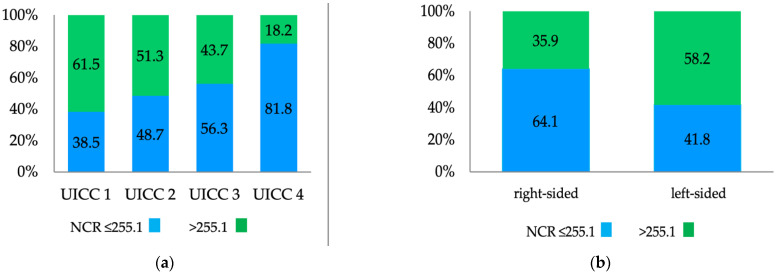
Clinical significance of the preoperative NCR in patients with colon cancer from the discovery cohort according to (**a**) the UICC classification: NCR levels were significantly decreased in a staged dependent manner; and (**b**) sidedness: significantly decreased NCR levels were observed in patients with right-sided colonic cancer.

We observed that low NCR levels were significantly associated with advanced tumors (Table 4), including undifferentiated histology (*p* < 0.043), lymph node metastasis (*p* < 0.041), distant metastasis (*p* < 0.006), and advanced UICC stage (*p* < 0.028) (Table 4, Figure 2). In addition, patients with elevated CEA concentrations presented with significantly decreased ratios of NCR compared with patients with normal CEA concentrations (*p* < 0.001, Table 4). Sex and patient age had no significant effects (Table 4). Moreover, right-sided tumors had a significantly lower NCR than left-sided tumors (*p* < 0.005, Figure 2).

### 3.4. Low Levels of NCR Were Associated with Poor Oncological Outcomes for Patients with Colon Cancer: Univariate and Multivariate Cox Regression Analysis of OS in the Discovery Cohort

In order to compare several established predictive parameters (NCR, CAR, LCR, ALI, sICAM, PNI, CEA, and NLR), hazard ratios were estimated in the discovery cohort for Overall Survival using Cox regression analysis (Table 5). NCR showed the highest hazard ratio of 4.33 (95% CI 2.34–8.00) compared to the other parameters, as well as the highest AUC in the ROC analysis (Figure 1, Table 3).

Multivariate analysis confirmed that age, sex, BMI, ASA, UICC and NCR were independent prognostic factors for poor OS (Table 6).

### 3.5. Survival Analysis

**Figure 3:**
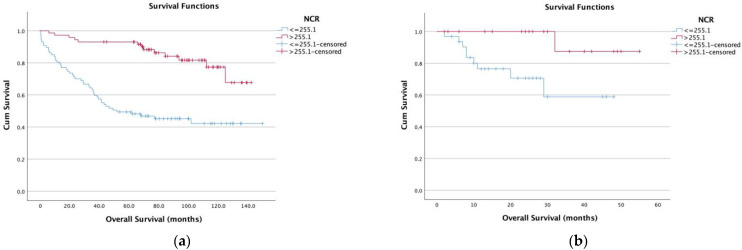
Prognostic effect of the NCR status on the OS of patients with colon cancer in the discovery cohort (**a**) and the validation cohort (**b**).

**Figure 4:**
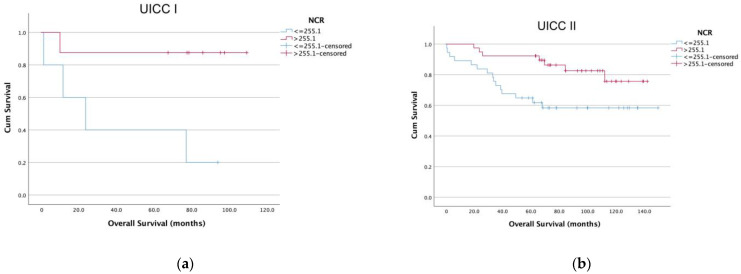

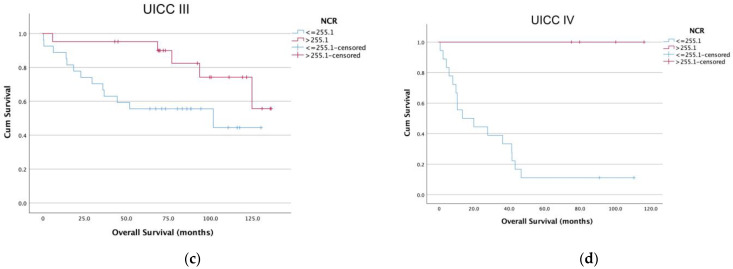
Prognostic effect of the NCR status on the OS of patients with colon cancer stratified according to UICC classification (**a**) Kaplan–Meier curves for OS based on UICC stage 1; (**b**) UICC stage II (**c**) UICC stage III and (**d**) UICC stage IV patients in the discovery cohort.

We performed a time-to-event analysis to evaluate the potential of NCR as a prognostic biomarker. Therefore, we generated Kaplan–Meier survival curves for OS split by NCR based on the optimal cutoff value using a ROC curve analysis with the Youden index in the discovery cohort (Figure 3a). Patients with low NCR levels had a significantly shorter OS (log-rank test, *p* < 0.001) than patients with high NCR levels (79.9 months ± 4.7 vs. 123.1 months ± 6.8). An analysis of all patients in the discovery cohort according to the UICC stage revealed a low NCR to be associated in every UICC stage with significantly shorter OS (Figure 4a–d). Low NCR was associated with a significantly shorter OS compared to high NCR in all UICC stages- UICC I: 41.5 months ± 16.5 versus 96.8 months ± 11.6 (*p* = 0.023); UICC II: 99.8 months ± 10 versus 124.2 months ± 6.2 (*p* = 0.017); UICC III: 79.3 months ± 10.2 versus 116.2 months ± 8 (*p* = 0.036); UICC IV: *p* = 0.006).

We confirmed these results by conducting a time-to-event analysis to evaluate the potential of the preoperative NCR using the same cutoff value as a prognostic biomarker in an external validation cohort (Figure 3b). Patients with lower NCR levels experienced a significantly shorter OS (log-rank test, *p* < 0.018) with a mean OS of 52.12 (±2.6) months for an NCR > 255.1 compared with 34.6 months (±3.7) for an NCR ≤ 255.1 in this external validation cohort. Furthermore, the multivariate Cox regression analysis also indicated that the NCR was an independent factor for predicting OS (HR 8.42, 95% CI 1.047–67.87, *p* = 0.045).

**Figure 5:**
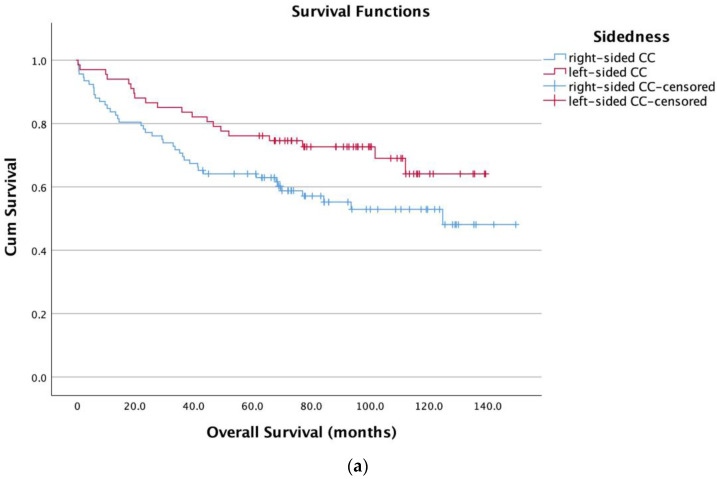

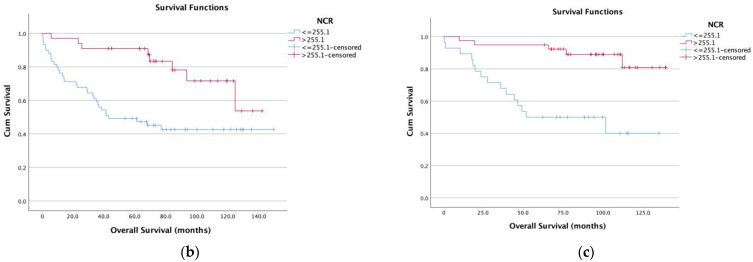
Kaplan–Meier curves for OS based on sidedness: right-sided and left-sided CC (**a**); Kaplan–Meier curves for OS based on NCR for right-sided colon cancer patients (**b**) and Kaplan–Meier curves for OS based on NCR for left-sided colon cancer patients in the discovery cohort (**c**).

Right- and left-sided CC patients with low levels of NCR had a significantly shorter OS (right 77.7 and left 78 months) compared to high levels of NCR (right-sided 116 and left-sided 126.7 months) (Figure 5). Patients with left-sided CC presenting with low NCR levels fared considerably worse than patients with right-sided CC.

## 4. Discussion

Predicting survival is essential since more radical treatment approaches might reduce the quality of life, which, on the other hand, is shortened by undertreatment. Despite the description of several new prognostic markers and multimodal treatment strategies, CRC still represents the third most common cause of cancer-related death. CRC prognosis is traditionally based on the TNM classification, the status of the resection margin, and specific histological and molecular features [5,19]. However, differences in outcomes and treatment response have been reported among patients within the same disease stage. Today, only patients with stage III CC are routinely offered adjuvant chemotherapeutic treatment. Fluorouracil monotherapy after surgical resection reduces the risk of death by 10–15% in patients with UICC stage III CC and by 20% when fluoropyrimidine-oxaliplatin therapy is applied [14,20,21]. Therefore, the portion of patients who actually benefit from adjuvant chemotherapy is approximately 20%, exposing up to 80% of patients to unnecessary toxicity. On the other hand, even with chemotherapy, the reported disease relapse rate is up to 30–50% [22]. When taken together, the therapeutic strategy currently used results in frequent undertreatment of patients with stage II disease (approximately 20% disease relapse [23,24,25]) and overtreatment of patients with stage III tumors. Thus, in the era of precision oncology, novel tools are needed for adequate prognostic staging, prediction of survival outcomes, and guiding multimodal therapy. With the emergence of immunological treatment options, the status of the immune system and inflammation severity have become the subjects of increasing focus of many studies [26]. The TNM staging system is limited in this regard. To the best of our knowledge, we are the first to report that the pretreatment combination of BMI and albumin levels along with CRP levels, which we defined as the NCR in the present study, is an independent indicator of a poor prognosis for patients with primary untreated colon cancer. The NCR reflects both the nutritional status, as represented by BMI and the serum albumin level, and the systemic inflammatory response, as represented by the CRP level. We confirmed our results in an independent external validation cohort.

First, we systematically investigated the prognostic effects of different established blood cell-based parameters (CAR, LCR, ALI, PNI, and NLR), CEA and other parameters (sICAM) in the discovery cohort. The impact of BMI on the prognosis of CRC, particularly CC, is discussed controversially [13]. We were previously able to document the effect of BMI on the long-term outcomes of 612 patients with rectal cancer and showed that underweight and excess body weight are associated with shorter OS and higher rates of distant metastasis [27]. The serum CRP level is an established inflammatory marker, and ratios incorporating this parameter have been reported as prognostic parameters for CRC [28,29]. However, numerous studies have analyzed the effects of systemic inflammatory or nutritional factors (or a combination of both) on CRC outcomes with inconclusive results [30,31,32]. One potential explanation is that statistical methods for reducing false-positive findings, such as bootstrapping, cross-validation, and consideration of multiple testing, were not conducted rigorously in many studies. Another potential explanation is the advantages and disadvantages of a prospective/retrospective study design. Furthermore, most of these outcome studies considered colon cancer and rectal cancer patients as a single disease entity.

In the present study, NCR was superior to the other prognostic indices and was a more reliable indicator of a poor prognosis for patients with colon cancer. The discriminatory ability of a biomarker is typically evaluated by performing sensitivity, specificity, and receiver operating characteristic curve (ROC) analyses. CEA is the most accepted and routinely used colorectal tumor marker for screening and predicting treatment response and survival. As a single marker, the CEA performance status predicting the outcome ranged from AUC 0.59 to 0.63 [33,34]. The NCR predicts the outcome with an AUC of 0.737 and a specificity of 86.79%. When included in multimarker panels, performance was greater than 0.8. In general, panels of biomarkers performed better than single markers [35]. Nevertheless, we propose that the translation of biomarkers to clinical implementation requires consideration of factors other than discriminatory ability and the combination of numerous markers. The optimal biomarker would be insensitive to variable preanalytical conditions, such as the time of day of sample collection or sample handling, which must be readily available and routinely used in clinical practice. In summary, using BMI, albumin levels, and CRP levels, our results provide a highly reproducible, easily obtainable, inexpensive, reliable, and practical biomarker for predicting the survival of patients with CC.

As mentioned, most studies regard CRC as one disease entity and do not consider the differences between the colon and rectum. We focused our attention on discerning blood-based markers governing colon cancer only. However, several issues must be addressed before integrating the NCR into treatment guidelines. First, our results must be validated in additional non-European cohorts. Second, the effects of microsatellite instability (MSI), mutational status, and NCR on patient prognosis must be investigated. A strength of our study is the prospective data collection and the two-step approach, with both a discovery and an independent validation cohort.

Our study also revealed interesting associations between the NCR and clinicopathological features. Lower preoperative NCR levels significantly correlated with undifferentiated histology, nodal involvement, and advanced UICC stage (Figure 2a). Interestingly, patients with left-sided tumors and low NCR scores performed significantly worse than patients with right-sided CC. In addition to the differentiation between the colon and rectum, tumor location (right-sided versus left-sided colon cancer) is widely accepted as a crucial factor determining disease progression and prognosis and influencing disease management [36,37]. Several publications have reported that older age, advanced T-stage, node-positive stage, and poor differentiation are more common in patients with right-sided colon cancer. Interestingly, right- and left-sided CC patients with low levels of NCR had a significantly shorter OS (right 77.7 and left 78 months) compared to high levels of NCR (right 116 and left 126.7 months). See Figure 5. In particular, left-sided CC patients with low levels of NCR fared considerably worse than right-sided CC patients. However, little is known about the impact of blood-based parameters on the prognosis of CC if sidedness is taken into consideration. Mazaki et al. evaluated the prognostic value of NLR by tumor sidedness and found that OS rates were significantly lower in patients with left-sided CC and high NLR levels [38].

Regarding the subgroup of patients with UICC stage II tumors, up to now, only patients with high-risk factors are candidates for adjuvant chemotherapy. Our data revealed that a low NCR was associated with a significantly shorter OS (Figure 4). Hence, the NCR might help clinicians select patient groups who would benefit from adjuvant treatment in stage II (low levels of NCR) or benefit from a reduced chemotherapy course in stage III (high levels of NCR). Therefore, further investigations of patients with stage II and III tumors are needed.

## 5. Conclusions

In summary, the therapeutic strategy used currently results in frequent undertreatment of patients with stage II CC and overtreatment of patients with UICC stage III tumors. We established the NCR as a sensitive prognostic marker of disease outcomes in patients with colon cancer. In all UICC stages, our newly developed biomarker is able to distinguish patients with better and worse prognoses. We, therefore, propose that NCR may serve as a supplement to the TNM staging system to optimize the risk stratification in CC patients towards personalized oncology.

## Figures and Tables

**Table 1:** Clinical characteristics of patients in the discovery cohort.

Demographics		*n* (%)
Age	years (median, range)	66 (27–91)
Sex	Male	103 (64.8)
	Female	56 (35.2)
Physical ASA status (American Society of Anesthesiologists)	1	25 (15.7)
	2	90 (56.6)
	3	44 (27.7)
BMI, kg/m²	<18.5	1 (0.6)
	18.5–24.9	46 (28.9)
	25–29.9	72 (21.4)
	30–34.9	34 (45.3)
	35–39.9	5 (3.1)
	≥40	1 (0.6)
UICC classification	Stage I	13 (8.2)
	Stage II	76 (47.8)
	Stage III	48 (30.2)
	Stage IV	22 (13.8)
Tumor location	Right-sided	92 (57.9)
	Left-sided	67 (42.1)
T-stage	T1	7 (4.4)
	T2	15 (9.4)
	T3	115 (72.3)
	T4	31 (19.5)
N-stage	N0	92 (57.9)
	N1	36 (22.6)
	N2	31 (19.5)
Histological differentiation	G1 well/G2 moderate	99 (62.3)
	G3 poor	60 (37.7)

**Table 2:** Characteristics of patients in the validation cohort.

Demographics		*n* (%)
Age	years (median, range)	68 (27–90)
Sex	Male	29 (54.7)
	Female	24 (45.3)
BMI, kg/m²	<18.5	2 (3.8)
	18.5–24.9	25 (47.2)
	25–29.9	20 (37.7)
	30–34.9	4 (7.5)
	35–39.9	1 (1.9)
	≥40	1 (1.9)
UICC classification	Stage I	0
	Stage II	19 (35.9)
	Stage III	13 (24.5)
	Stage IV	21 (39.6)

**Table 3:** Receiver operating characteristic (ROC) curve analysis to evaluate the predictive value of inflammatory biomarkers for OS in the discovery cohort.

Variables	AUC	Cutoff	Sensitivity	Specificity	95% CI (Upper-Lower Limits)
NCR	0.737	>255.1	61.3%	86.8%	0.803–0.661
CAR	0.722	≤0.1	59.4%	84.9%	0.790–0.645
LCR	0.707	>0.2	66.0%	77.4%	0.776–0.629
ALI	0.679	>252.2	83.7%	47.2%	0.75–0.6
sICAM	0.643	≤229.1	46.2%	81.1%	0.718–0.564
PNI	0.674	>42.6	65.1%	67.9%	0.746–0.595
NLR	0.641	≤3.54	70.8%	58.5%	0.716–0.561
CEA	0.680	5.0	74.5%	56.6%	0.8–0.6

**Table 4:** The relationship between clinicopathological variables and NCR in patients with colon cancer in the discovery cohort. (ECOG PS: Eastern Cooperative Oncology Group performance status).

Parameter	Groups	Total *n* (%)	NCR *n* ≤ 255.1	NCR *n* > 255.1	*p* Value
Age	<66 years	79 (49.7)	43	36	0.942
	>66 years	80 (50.3)	44	36	
Sex	Male	103 (64.8)	60	43	0.225
	Female	56 (35.2)	27	29	
ECOG PS	0	93 (58.5)	38	55	**<0.001**
	1	42 (30.2)	25	17	
	2	11 (6.9)	11	0	
	3/4	13 (8.2)	12	1	
Tumor location	Right-sided	92 (57.9)	59	33	**0.005**
	Left-sided	67 (42.1)	28	39	
Histological differentiation	G1/G2	99 (62.3)	48	51	**0.043**
	G3	60 (37.7)	39	21	
N-stage	N0	92 (57.9)	44	48	**0.041**
	N+	67 (42.1)	43	24	
Inflammatory stromal reaction	moderate	62 (39)	37	46	**0.004**
	pronounced	77 (48.4)	25	31	
	unknown	20 (12.6)	4	16	
Distant metastasis	no	137 (86.2)	69	68	**0.006**
	yes	22 (13.8)	18	4	
UICC classification	Stage I	13 (8.2)	5	8	**0.028**
	Stage II	76 (47.8)	37	39	
	Stage III	48 (30.2)	27	21	
	Stage IV	22 (13.8)	18	4	
CEA	≤5	102 (64.2)	46	41	**0.001**
	>5	57 (35.8)	56	16	

**Table 5:** Hazard Ratios for various predictive parameters (Cox regression analysis of the OS of patients with colon cancer in the discovery cohort).

Variables	Categorical	Hazard Ratio	95% CI Low	95% CI High	*p* Value
NCR	>255.1	4.33	2.34	8.00	<0.001
CAR	≤0.11	3.78	2.08	6.88	<0.001
LCR	>0.24	3.52	2.02	6.12	<0.001
ALI	>252.2	3.00	1.80	5.00	<0.001
sICAM	≤229.1	2.10	1.48	4.93	0.001
PNI	>42.6	2.56	1.52	4.31	<0.001
CEA	>5.0	2.44	1.47	4.04	0.001
NLR	≤3.54	2.06	1.25	3.41	0.005

**Table 6:** Multivariate analysis of clinicopathological variables for OS in patients with CC in the discovery cohort. (ECOG PS: Eastern Cooperative Oncology Group performance status).

	Univariate Analysis			Multivariate Analysis		
	HR	95% CI	*p* Value	HR	95% CI	*p* Value
Age	1.037	1.010–1.065	0.006	1.043	1.012–1.076	**0.006**
Sex	2.501	1.353–4.620	**0.003**	4.280	2.177–8.417	**<0.001**
Male						
Female						
BMI	0.944	0.887–1.004	0.067	0.922	0.862–0.986	**0.018**
ASA						
1			**<0.001**			**0.025**
2	1.622	0.626–4.205	0.320	1.210	0.429–3.412	0.719
3	4.473	1.724–11.607	0.002	2.526	0.846–7.540	0.097
ECOG						
0			**0.002**			
1	1.665	0.916–3.024	0.094			
2	3.777	1.629–8.754	0.002			
3	3.510	1.588–7.757	0.002			
4	5.231	0.703–38.932	0.106			
T-stage						
T1			0.11			
T2	1.229	0.238–6.338	0.193			
T3	1.241	0.300–5.136	0.139			
T4	2.703	0.604–12.094	0.018			
N-stage						
N0			<0.001			
N1	1.111	0.565–2.186	0.760			
N2	3.115	1.763–5.506	<0.001			
M-stage						
M0			<0.001			
M1a (one organ)	1.834	0.727–4.629	0.199			
M1b (several organs)	3.933	0.943–16.402	0.060			
M1c (peritoneum)	7.779	3.699–16.360	<0.001			
Tumor location	1.743	1.020–2.978	**0.042**			
Right						
Left						
Lymphocytes	0.989	0.639–1.531	0.961			
Thrombocytes	1.001	0.999–1.004	0.261			
Albumin	0.89	0.853–0.945	**<0.001**			
CRP	1.009	1.000–1.017	**0.041**			
CEA	1.001	1.000–1.002	**0.007**			
UICC						
Stage I			**<0.001**			**<0.001**
Stage II	0.631	0.238–1.672	0.354	0.375	0.131–1.076	0.068
Stage III	0.871	0.323–2.352	0.786	0.451	0.152–1.337	0.151
Stage IV	2.673	0.976–7.318	0.056	1.865	0.587–5.920	0.290
NCR	4.325	2.337–8.003	**<0.001**	3.305	1.628–6.709	**0.001**
≤255.1						
>255.1						

## Data Availability

Data supporting this research are included in the published article.

## Supplementary Materials

The online version contains supplementary material available online: https://www.mdpi.com/article/10.3390/diagnostics13010116/s1. Figure S1: ROC curves for NCR, CAR, ALI, PNI, CEA, sICAM, NLR, and LCR.

## Abbreviations

NCR: nutritional CRP ratio; CRC: colorectal cancer; CC: colon cancer; CEA: carcinoembryonic antigen; ICAM-1: intercellular adhesion molecule; LFA-1: leukocyte integrin lymphocyte function-associated antigen; LCR: lymphocyte-to-C-reactive Protein ratio; NLR: neutrophil-to-lymphocyte ratio; PLR: platelet-to-lymphocyte ratio; BMI: body mass index; PNI: prognostic nutritional index; ALI: advanced lung cancer inflammation index; CAR: the C-reactive protein to albumin ratio; ROC: receiver operating characteristic; AUC: area under the ROC curve; KM: Kaplan–Meier; HR: hazard ratio; CI: confidence interval; UICC: Union Internationale Contre le Cancer; ECOG PS: Eastern Cooperative Oncology Group performance status; ASA: American Society of Anesthesiologists.
