# Supplementary figures and images for: NCR, an Inflammation and Nutrition Related Blood-Based Marker in Colon Cancer Patients: A New Promising Biomarker to Predict Outcome
Source: Diagnostics (Basel). 2022 Dec 30;13(1):116. doi: 10.3390/diagnostics13010116 (PMC9818830; doi:10.3390/diagnostics13010116)

Supplementary figures:

Figure S1

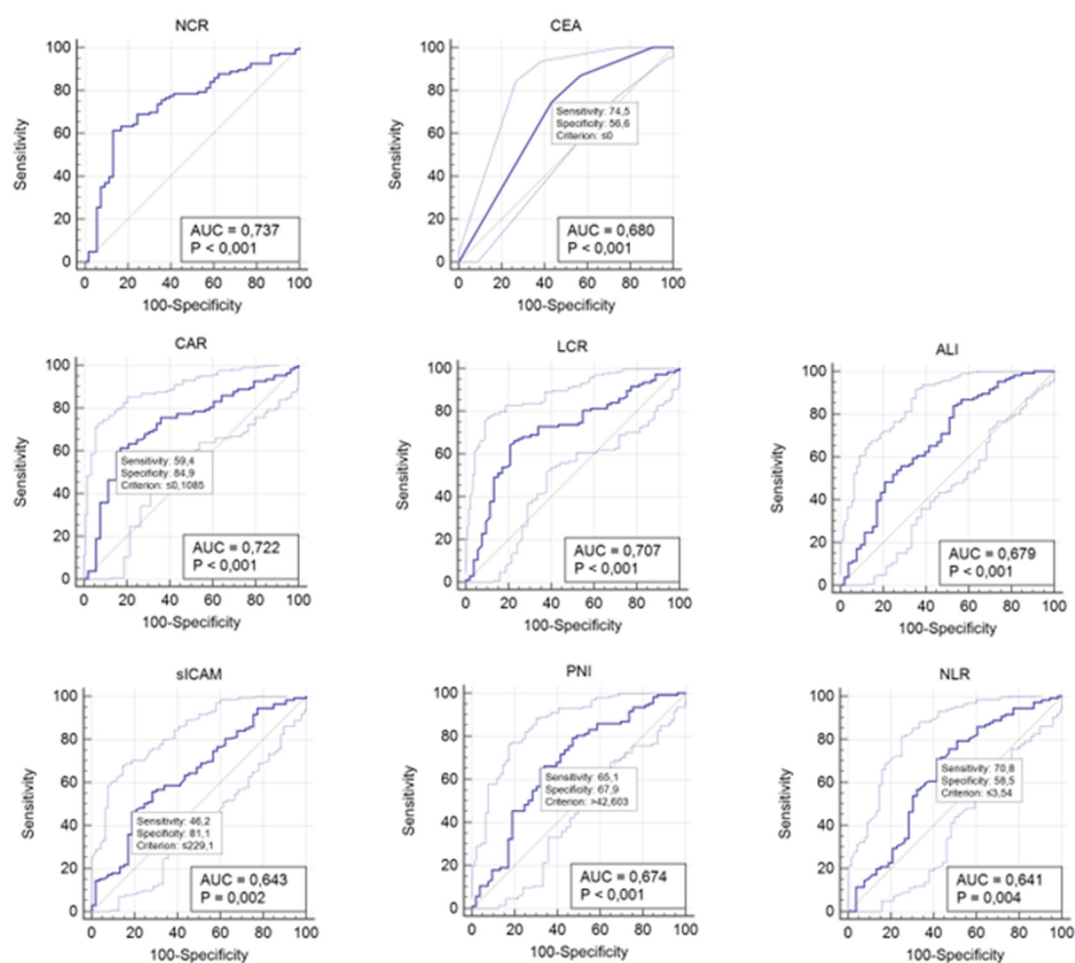

Figure S1: ROC curves for NCR, CAR, ALI, PNI, CEA, sICAM, NLR, and LCR.

Supplement: Supplementary file 1 [file diagnostics-13-00116-s001.zip › diagnostics-2094662-supplementary.pdf]
